# Epithelial-specific ERBB3 deletion results in a genetic background-dependent increase in intestinal and colon polyps that is mediated by EGFR

**DOI:** 10.1371/journal.pgen.1009931

**Published:** 2021-11-29

**Authors:** Carolina Mantilla Rojas, Michael P. McGill, Anna C. Salvador, David Bautz, David W. Threadgill

**Affiliations:** 1 Interdisciplinary Program in Genetics, Texas A&M University, College Station, Texas, United States of America; 2 Department of Molecular and Cellular Medicine, Texas A&M University, College Station, Texas, United States of America; 3 Department of Nutrition, Texas A&M University, College Station, Texas, United States of America; 4 Department of Genetics, North Carolina State University, Raleigh, North Carolina, United States of America; 5 Department of Biochemistry & Biophysics and Department of Nutrition, Texas A&M University, College Station, Texas, United States of America; National Cancer Institute, UNITED STATES

## Abstract

ERBB3 has gained attention as a potential therapeutic target to treat colorectal and other types of cancers. To confirm a previous study showing intestinal polyps are dependent upon ERBB3, we generated an intestinal epithelia-specific ERBB3 deletion in C57BL/6-*Apc*^*Min/+*^ mice. Contrary to the previous report showing a significant reduction in intestinal polyps with ablation of ERBB3 on a B6;129 mixed genetic background, we observed a significant increase in polyp number with ablation of ERBB3 on C57BL/6J compared to control littermates. We confirmed the genetic background dependency of ERBB3 by also analyzing polyp development on B6129 hybrid and B6;129 advanced intercross mixed genetic backgrounds, which showed that ERBB3 deficiency only reduced polyp number on the mixed background as previously reported. Increased polyp number with ablation of ERBB3 was also observed in C57BL/6J mice treated with azoxymethane showing the effect is model independent. Polyps forming in absence of ERBB3 were generally smaller than those forming in control mice, albeit the effect was greatest in genetic backgrounds with reduced polyp numbers. The mechanism for differential polyp number in the absence of ERBB3 was through altered proliferation. Backgrounds with increased polyp number with loss of ERBB3 showed an increase in cell proliferation even in non-tumor epithelia, while backgrounds showing reduced polyp number with loss of ERBB3 showed reduced cellular proliferation. Increase polyp number caused by loss of ERBB3 was mediated by increased epidermal growth factor receptor (EGFR) expression, which was confirmed by deletion of *Egfr*. Taken together, this study raises substantial implications on the use of ERBB3 inhibitors against colorectal cancer. The prediction is that some patients may have increased progression with ERBB3 inhibitor therapy, which is consistent with observations reported for ERBB3 inhibitor clinical trials.

## Author summary

Targeted cancer therapeutics that are efficacious in pre-clinical studies do not always translate to the clinic. This can be due to the homogenous pre-clinical models not representing heterogeneous patient populations. Small molecular inhibitors to ERBB3 for colorectal cancer efficiently inhibited tumorigenesis in pre-clinical studies, but in clinical trials failed to show efficacy and even trended toward enhancing cancer growth. Using two mouse colorectal cancer models with variable the genetic backgrounds, we show that epithelial deletion of ERBB3 can result in outcomes ranging from inhibition to enhanced tumor growth. Molecular profiling implicated EGFR as a likely mediator of enhanced tumor growth in the absence of ERBB3. Mice with epithelial deletion of both EGFR and ERBB3 were used to show that the background dependency of ERBB3 is mediated by EGFR. These results show that genetic context of cancer therapeutics can have profound implications on translating pre-clinical results into the clinic.

## Introduction

Members of the EGFR/ERBB receptor tyrosine family have been studied intensively as therapeutic targets against a variety of cancers [1]. Because of its unique characteristics, attention has focused on ERBB3, which shares core structural domains with other ERBB receptors [2]. However, ERBB3 cannot auto-phosphorylate due to a lack of intrinsic kinase activity distinguishing it from other ERBB receptors [3], although recent evidence suggests the ERBB3 kinase may still be important during heterodimerization with other ERBB receptors [4]. Tyrosine-phosphorylated ERBB3 provides an efficient docking site for downstream adaptor proteins. Notably, ERBB3 has nine docking sites for the p85 subunit of phosphatidylinositol 3-kinase (PI3K) giving it the highest binding affinity for PI3K among the ERBB receptors [5]. Because of the high PI3K binding capacity, protein kinase B (AKT) signaling is strongly activated by ERBB3, which results in an oncogenic stimulus frequently implicated in many cancers and during therapeutic resistance [6].

Although most clinical focus has been on EGFR and ERBB2 due to their aberrant activation in many human malignancies [7], overexpression of ERBB3 often co-occurs with EGFR or ERBB2 in many types of cancers such as breast [8,9], colorectal (CRC) [2,10], gastric [11,12], ovarian [13], and pancreatic [14]. In ERBB2-driven cancers, ERBB3 enhances cancer progression, usually by PI3K/AKT pathway activation, by functioning as a heterodimer partner [15,16]. For these cancers in particular, ERBB3 inhibition may be required to effectively eradicate cancerous cells [17]. Similar to ERBB2, EGFR can be efficiently coupled to the PI3K/AKT pathway by heterodimerization with ERBB3. This is most recognized in non-small cell lung cancers (NSCLC) sensitive to EGFR inhibitors [18]. ERBB3-dependent activation of PI3K/AKT can underlie acquired resistance to EGFR inhibitors in a subset of NSCLC patients that is due to amplification of MET [18]. Blocking of ERBB3 genetically or pharmacologically showed promising preclinical results [19–23], including as a prophylactic vaccination target for at-risk CRC populations [24]. However, in the clinic, surprisingly little evidence for efficacy was observed against CRC [25,26].

A major weakness in most preclinical studies is the lack of attention to variation in genetic background and how this might impact perceived efficacy. This is particularly of concern for clinical trials that are designed primarily to observe off-target toxicities in phase I and progression-free survival in phase II/II. Results of clinical trials with ERBB3 inhibitors against CRC have been disappointing, and possibly even detrimental. The FOCUS4-D trial with AZD8931, a pan-ERBB inhibitor against EGFR, ERBB2, and ERBB3, showed no efficacy, and even trended to shorter progression-free survival over the placebo group [27]. Similarly, comparison of duligotuzumab, an EGFR/ERBB3 dual inhibitor, showed no benefit over the EGFR inhibitor cetuximab and also trended to a lower objective response rate [28]. These results raise concerns whether inhibiting ERBB3 during CRC treatment may actually be detrimental in some patients, masking those cases that might benefit in clinical trials.

We previously generated a conditional *Erbb3* allele (*Erbb3*^*tm1*.*1Dwt*^ referred as *Erbb3*^*f*^) to perform tissue-specific ablation of ERBB3, which was used to show ERBB3-dependent signaling during intestinal tumorigenesis [23]. On a mixed C57BL/6J and 129S1/SvImJ genetic background (B6;129), we showed that ERBB3 is essential for tumor development. In light of the conflicting clinical trials with ERBB3 inhibition, we attempted to confirm the previous results using a congenic C57BL/6J genetic background. Surprisingly, in two mouse models of human CRC, the *Apc*^*Min*^ model of spontaneous intestinal tumorigenesis and a model of colitis-associated colorectal (CAC) tumorigenesis induced by the colon-selective carcinogen azoxymethane (AOM), ERBB3 deficiency dramatically increased tumor multiplicity. Polyps arising in the absence of ERBB3 also showed increased transcript levels of *Erbb4* and *Egf*, consistent with previous results on the B6;129 mixed background [23]. To confirm if the original results were due to the 129S1/SvImJ background, we crossed the conditional *Erbb3* allele to the 129S1/SvImJ background and found that ERBB3-deficiency results in a dramatic reduction in polyp multiplicity in the *Apc*^*Min/+*^ mouse model, similar to the original observations. In contrast, on a B6129 hybrid background, the lack of ERBB3 has no impact on tumorigenesis. Based on the known background sensitivity of EGFR, deletion of both *Egfr* and *Erbb3* on a C57BL/6J background showed a dramatic decrease in polyp number indicating the tumor increase with loss of ERBB3 on the C57BL/6J background was at least partially mediated by EGFR. Together these results suggest that ERBB3 inhibitor response in CRC patients could range from highly efficacious to promoting progression depending on the genetic background of the patient, requiring a re-evaluation of how clinical trials are monitored and analyzed.

## Results

### Requirement for ERBB3 during intestinal polyp development is genetic background-dependent

**Fig 1:**
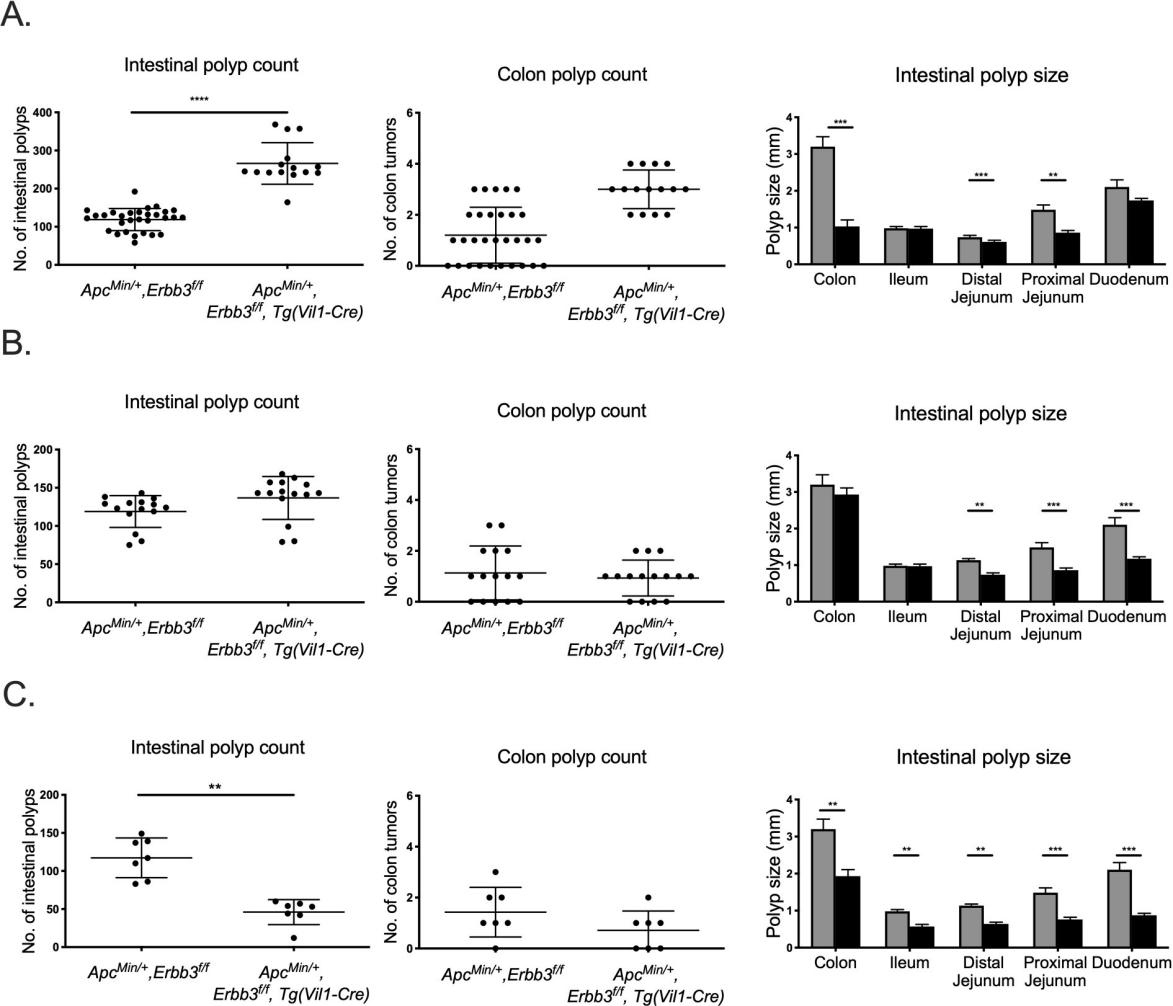
Genetic background alters effect of ERBB3 deficiency on intestinal polyp development. Polyp number and sizes in mice lacking ERBB3 in intestinal epithelium and from *Apc*^*Min*/+^ mice on (A) C57BL/6J; (B) B6129 hybrid background; and (C) B6;129 mixed backgrounds. Each dot represents polyp number in each three-month-old mouse. Grey bars represent mean polyp size in *Erbb3* wildtype mice and black bars represent mean polyp size of *Erbb3* intestinal epithelia-specific knockout mice. * p-value<0.05, ** p-value < 0.01, *** p-value < 0.001, **** p-value < 0.0001.

To evaluate the role of ERBB3 signaling during intestinal tumorigenesis, we used the *Apc*^*Min/+*^ mouse model with intestinal epithelium specific deletion of *Erbb3*, using *Vil1-Cre* and conditional knockout allele of *Erbb3* (C57BL/6J-*Apc*^*Min/+*^, *Erbb3*^*f/f*^, *Tg(Vil1-Cre)*) and wild-type littermate controls (C57BL/6J-*Apc*^*Min/+*^, *Erbb3*^*f/f*^). At three months of age, all *Apc*^*Min/+*^ mice on a C57BL/6J background (n = 30 ERBB3 wild-type; n = 15 ERBB3 deficient) developed visible polyps (>0.3mm in diameter) in the small intestine regardless of ERBB3 genotype (Fig 1A). In dramatic contrast to a previous study analyzing ERBB3 deficiency on a B6;129 mixed genetic background [23], the number of intestinal polyps in *Apc*^*Min/+*^ mice lacking ERBB3 were significantly increased compared with that in *Apc*^*Min/+*^ controls. This ERBB3-dependent increase in the number of small intestine polyps was observed in all regions of the small intestine, and trended in the same direction in the colon. Whereas 66% of the *Apc*^*Min/+*^ control mice developed at least one colon polyp, all *Apc*^*Min/+*^ lacking ERBB3 developed at least two colon polyps. Altogether, these results demonstrated that epithelial-specific ERBB3 signaling is important during intestinal tumorigenesis in *Apc*^*Min/+*^ mice. This current study used a different genetic background (C57BL/6J) from the previous study (B6;129 mix) that reported reduced intestinal polyps with loss of ERBB3, suggesting a genetic background-dependent effect of ERBB3 on the development of intestinal polyps.

To assess if genetic background differences were responsible for the contradicting results when ERBB3 is removed, a B6129 hybrid population lacking *Erbb3* in the intestinal epithelium (*Apc*^*Min/+*^, *Erbb3*^*f/f*^, *Tg(Vil1-Cre)*) was compared to littermate controls with wild-type *Erbb3* (*Apc*^*Min/+*^, *Erbb3*^*f/f*^*)*. In the B6129 hybrid background, there was no significant difference in the number of intestinal and colon polyps in the absence of ERBB3 (Fig 1B). When to the 129S1/SvImJ background to generate an advanced intercross mixed background, a decrease in the number of intestinal polyps was observed compared to wild-type littermates, consistent with the previous study that used the B6;129 mixed genetic background (Fig 1C). Despite differences in tumor number, the size of ERBB3-deficient intestinal polyps was reduced on all three genetic backgrounds, albeit only significantly across all regions of the intestinal tract in the B6;129 mixed background that also showed a significant decrease in polyp number. Histological analysis of size-matched polyps did not reveal morphological differences related to ERBB3 genotype.

### ERBB3 signaling prevents apoptosis and, dependent on genetic background, alters proliferation in *Apc*^*Min/+*^ polyps

**Fig 2:**
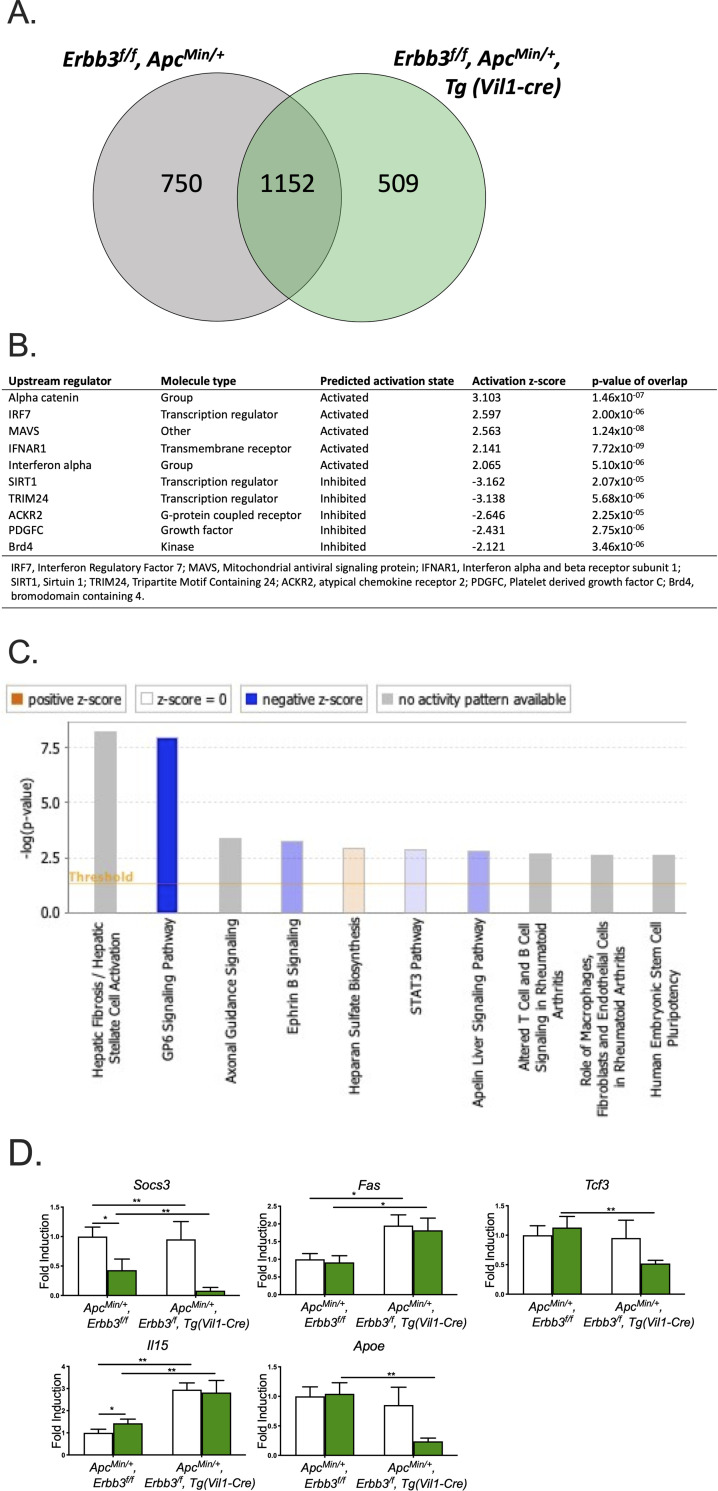
Transcriptomic analysis of ERBB3-deficient colonic polyps from C57BL/6J mice. (A) Venn diagram of differentially expressed genes between wild-type and ERBB3-deficient colonic polyps compared to adjacent normal epithelium. (B) Top upstream regulators characterizing ERBB3*-*deficient intestinal polyps. (C) Significant canonical pathways identified from IPA to be deregulated in ERBB3-deficient intestinal polyps. (D) Validation of differentially expressed genes in ERBB3-deficient intestinal polyps. White bars represent mean level of transcripts in normal tissue adjacent to polyps; green bars represent mean level of transcripts in the colon polyps with different *Erbb3* genotypes. * p-value < 0.05, ** p-value < 0.01, *** p-value < 0.001, **** p-value < 0.0001.

To determine the mechanism underlying the increase in intestinal polyp number in the absence of ERBB3 on a C57BL/6J background, transcript data from colon polyps lacking ERBB3 was compared to polyps from littermates with wild-type levels of ERBB3 revealing 509 genes characteristic of ERBB3-deficient colon polyps (Fig 2A). Ingenuity pathway analysis (IPA) predicted several upstream regulators (Fig 2B) and canonical pathways (Fig 2C) that characterize ERBB3-deficient polyps including downregulation of *MAPK1* (S1 Fig). Differentially expressed genes involved in these cellular functions (*Socs3*, *Fas*, *Tcf3*, *IL15*, *Apoe*) were validated by quantitative PCR (Fig 2D). These genes have been associated to CRC progression affecting different pathways. Low expression of *Socs3* [29] and *Tcf3* [30] have been associated with the occurrence and progression of CRC, while upregulation of *Fas* [31] and I*l15* [32] is associated with malignant potential in colon cancer cells by promoting tumor motility and metastasis. The role of *Apoe* has been associated with DNA synthesis, cell proliferation, angiogenesis and metastasis, so the aberration of these functions may lead to tumorigenesis and progression. However, the role of *Apoe* in CRC progression remains unclear [33].

**Fig 3:**
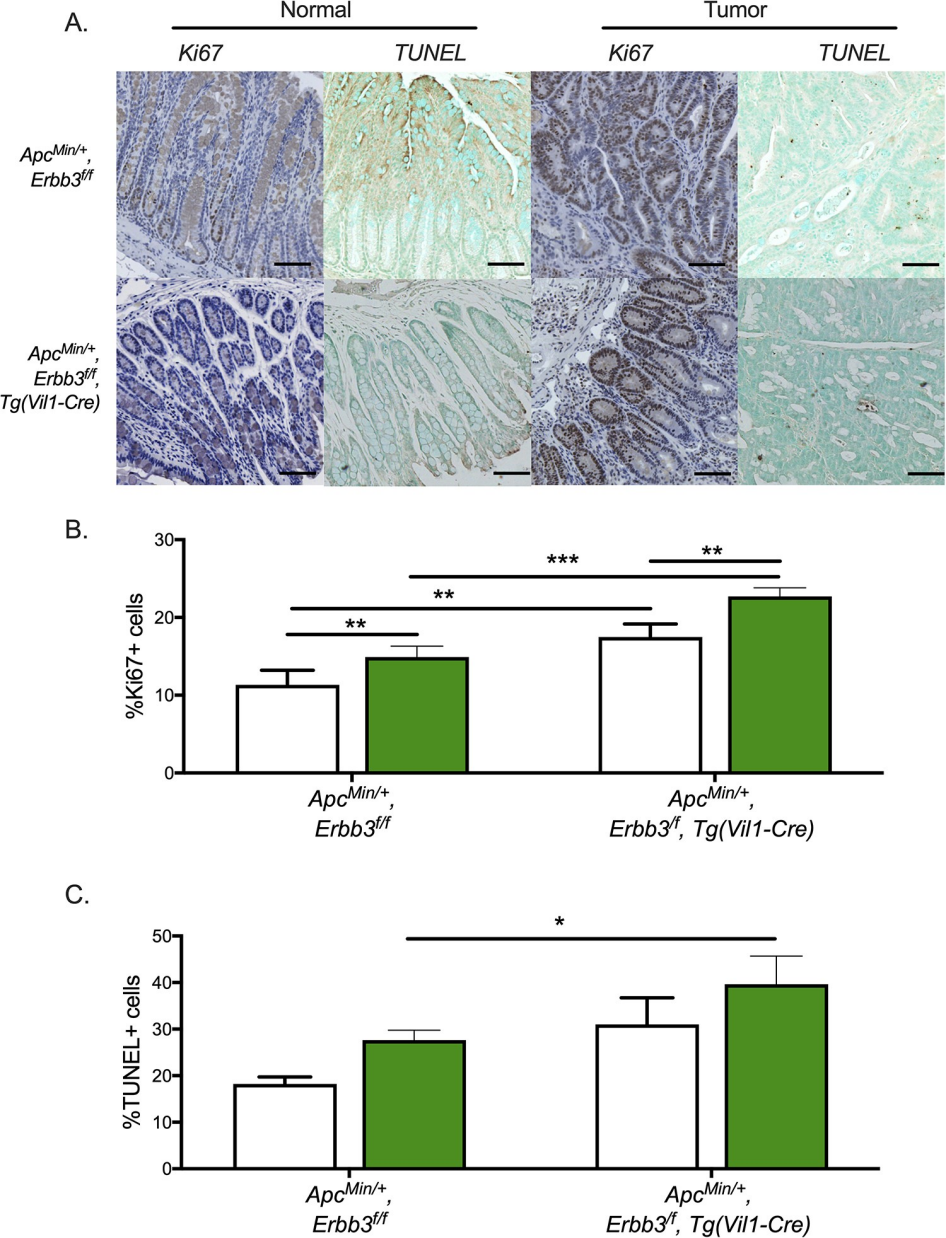
Cellular effects of ERBB3-deficiency on a C57BL/6J background. (A) Staining for proliferative (Ki67) and apoptotic (TUNEL) cells in polyps and adjacent normal tissue. Quantitative comparison of (B) Ki67 and (C) TUNEL counts in tumor and adjacent normal intestinal tissue. White, adjacent normal; green, polyp. p-value < 0.05, ** p-value < 0.01, *** p-value < 0.001.

Transcriptomic analysis using IPA also revealed increased mRNA levels of genes associated with proliferation and apoptosis mechanisms. The proliferative and apoptotic rates within *Apc*^*Min*/+^ polyps were measured to validate the IPA prediction. Staining for Ki67 revealed a slight increase in proliferative cells in polyps as well as normal epithelium from ERBB3-deficient mice when compared to samples with normal levels of ERBB3 (Fig 3A and 3B). The apoptotic rate in polyps was measured using a TUNEL assay, and a significant increase in the number of TUNEL-positive cells was observed in polyps from ERBB3-deficient mice compared with polyps from littermates with normal levels of ERBB3 (Fig 3A and 3C).

The previous study investigating the role of ERBB3 in intestinal polyp development using a B6;129 mixed genetic background indicated elevated apoptosis in ERBB3-deficient polyps, but, unlike on the C57BL/6J genetic background, the number of proliferative cells in normal epithelium was decreased and restricted to the base of the crypts [23]. Together, these results suggest that the increase in the number of polyps in C57BL/6J-*Apc*^*Min/+*^ mice lacking ERBB3 is likely due to increased proliferation in normal epithelium, while the reduced polyp number on the B6;129 mixed background is due to reduced proliferation in normal epithelium. These studies also showed that irrespective of ERBB3 genotype, TUNEL-positive cells were increased in *Apc*^*Min/+*^ polyps with no significant difference between genetic backgrounds groups. An important consideration is that although an increase in the number of TUNEL-positive cells was observed in ERBB3-deficient polyps compared to littermate controls on both C57BL/6J and B6;129 mixed genetic backgrounds, the reduction in polyp size varied across genetic backgrounds when there are no changes or an increase in polyp number, while showing consistent reduction in size across all regions of the intestinal tract in the B6;129 mixed background that has a significant reduction in polyp numbers.

### Loss of ERBB3 increases colon polyp number but does not alter size in the AOM model

**Fig 4:**
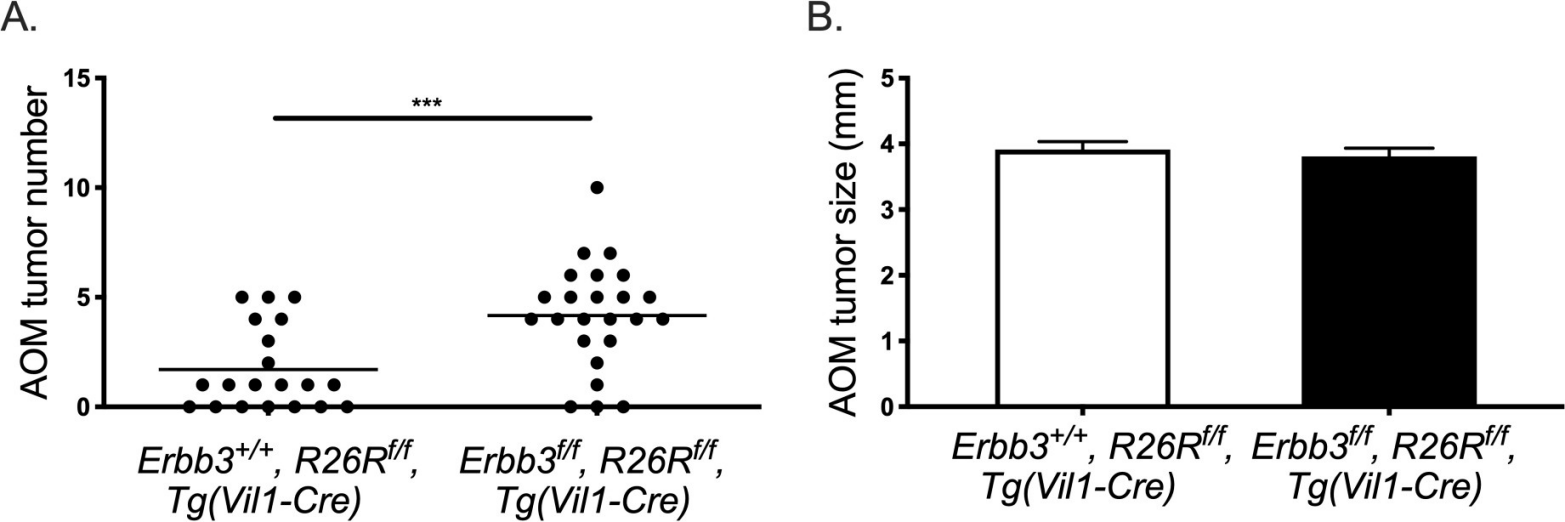
ERBB3-deficiency increases AOM-induced colonic polyp development in C57BL/6J mice. Colonic polyp (A) multiplicity and (B) size in AOM-treated mice. Each dot represents the number of colon polyps after induction by AOM. White bars represent the mean tumor size of colon polyps with normal ERBB3 and black bars represent the mean tumor size of ERBB3-deficient colon polyps induced by AOM. * p-value < 0.05, ** p-value < 0.01, *** p-value < 0.001, **** p-value < 0.0001.

To determine whether the genetic background dependency on ERBB3 signaling was model dependent, the effect of ERBB3-deficiency was also investigated on a mouse model of colitis-associated colorectal tumorigenesis (CAC) induced by the colon-selective carcinogen azoxymethane (AOM). Similar to the C57BL/6J-*Apc*^*Min/+*^ model, a significant increase in AOM-induced colon polyp multiplicity was observed in the absence of ERBB3 on the C57BL/6J background (Fig 4). More than half (53%) of mice with normal levels of ERBB3 treated with AOM developed one or more colonic tumors, with an average of 1.7 tumors per mouse. ERBB3-deficiency increased susceptibility to AOM. Approximately 90% of mice with intestinal deficiency of ERBB3 developing colonic tumors, with an average of 4.2 tumors per mouse. In contrast to the size-effect on residual polyps observed in the *Apc*^*Min/+*^ mouse model, there was no significant difference in the size of AOM-induced colonic polyps between the two groups (3.65 ± 1.6 vs. 3.58 ± 1.1; p = 0.92). These findings indicate that, dependent on genetic background, loss of ERBB3 can contribute to increased intestinal and colonic tumors irrespective of the model.

### Effect of ERBB3 on Apc^Min/+^ polyp numbers is dependent on EGFR

**Fig 5:**
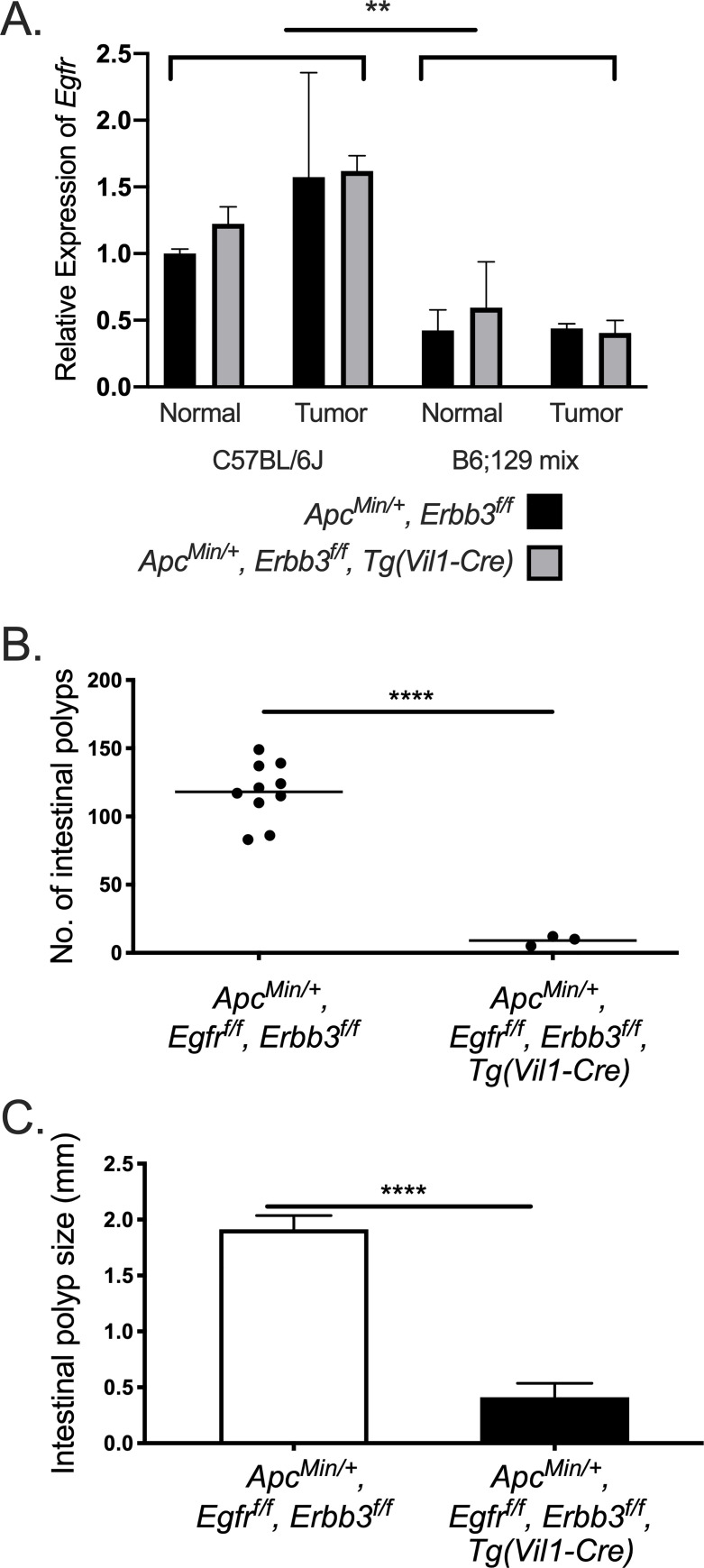
ERBB3-dependent polyp number increase in C57BL/6J-*Apc*^*Min/+*^ mice is dependent on EGFR. Expression of *Egfr* in normal and tumor tissue from mice on with and without ERBB3 on both backgrounds (A). *Egfr*, *Erbb3* double mutation reduces the (B) number and (C) size of intestinal polyps in C57BL/6J-*Apc*^*Min/+*^ mice. Each dot represents the number of polyps across the intestinal tract. White bars represent mean tumor size of polyps with normal levels of EGFR and ERBB3 mice and black bars represent mean tumor size in EGFR and ERBB3 double deficient mice. ** p-value<0.01, *** p-value<0.001, **** p-value<0.0001.

Deletion of EGFR results in a classic genetic background dependent effect on resulting phenotypes [34–36], including tumorigenesis in the *Apc*^*Min/+*^ and AOM models [37]. To determine if the strain-dependent effect of ERBB3-deficiency is mediated by EGFR, expression of *Egfr* was analyzed by qPCR in C57BL/6J and B6;129 mixed backgrounds (Fig 5A). Although there was no statistically significant difference between normal epithelium and tumor on either background, there was a significant increase in *Egfr* expression on C57BL/6J compared to the B6;129 mixed background, which was consistent with EGFR detected by IHC (S2 Fig). This differential expression is trans-mediated since C57BL/6J and 129S1/SvImJ are identical-by-descent at *Egfr*.

To test if increased *Egfr* contributed to increased tumor number on the C57BL/6J background, intestinal-specific double mutants were generated by deletion of EGFR and ERBB3 in C57BL/6J-*Apc*^*Min/+*^ mice (*Apc*^*Min/+*^, *Erbb3*^*f/f*^, *Egfr*^*f/f*^, *Tg(Vil1-Cre)*) (Fig 5B and 5C). Fewer double mutant mice were obtained than expected based on the cross, with five out of 100 animals being double mutant and only three surviving to three months of age. Double mutant mice appeared to be anemic, denoted by clear white paws and tail, although the mechanism underlying this phenotype is unknown. When compared to wild-type littermates, significantly fewer and smaller intestinal polyps were found in *Egfr*, *Erbb3* double mutant mice, similar to the effect of *Erbb3* loss on the B6;129 mixed background. Taken together, these results show that loss of ERBB3 on some backgrounds increases tumorigenesis through an EGFR-dependent mechanism.

## Discussion

ERBB3 is the only member of the ERBB family that lacks intrinsic kinase activity, although evidence shows that tumor phenotypes can be modulated by activation of ERBB3-dependent pathways [38,39]. We examined intestinal polyp development in the absence of ERBB3 activity using intestinal epithelial-specific deletion of *Erbb3*. Previous analysis using the *Apc*^*Min/+*^ mouse model reported that ERBB3 deficiency significantly decreases mean polyp number and size on a B6;129 mixed genetic background. When we attempted to confirm these results on an isogenic C57BL/6J background, absence of ERBB3 actually has the opposite effect, with a significant increase in the number of polyps. ERBB3-deficiency on a B6129 hybrid background had no effect on polyp number, while a B6;129 mixed background confirmed the previous mixed background results with a significant reduction in the number of polyps. This result demonstrates a striking strain-dependent effect of ERBB3 on the development of intestinal polyps. Although background strongly influences polyp number in the absence of ERBB3, previous prophylactic vaccination studies using a specific anti-ERBB epitope in C57BL/6J-*Apc*^*Min/+*^, which resulted in reduced polyp number [24], indicates that the effect is more complicated and different methods of inhibiting ERBBs may have varying responses.

Due to this background dependency, which is reminiscent of the background sensitivity of EGFR on polyp induction [37], we further show that the enhanced polyp number on the C57BL/6J background can be eliminated in a double deficiency of EGFR and ERBB3 in the intestinal epithelium. Other backgrounds may have different compensatory mechanisms when ERBB3 is inhibited based on the heterogenous compensatory mechanisms underlying loss of EGFR [35]. The role of ERBB3 in colonic tumor development was confirmed using a distinctly different model, the AOM mouse model of CRC. These results raise an intriguing dilemma for therapeutic development, the possibility that therapeutic interventions designed to reduce growth of cancers may in fact enhance growth in some patients. Patients that progress under therapy are usually assumed to be non-responders. However, some cancers may have accelerated growth caused by the therapy, which has important implications for inhibitor therapies in the clinic.

Despite the genetic background dependency on polyp development, ERBB3-deficiency generally decreased the average size of *Apc*^*Min/+*^ polyps independent of the genetic background, albeit the effect was inconsistent across intestinal regions on backgrounds where ERBB3 deficiency did not reduce polyp number. The effect of reduced in polyp size with ERBB3 loss is opposite to the effect previously reported for loss of EGFR. Previously, Roberts et al., demonstrated that reduced EGFR activity promoted the development of larger intestinal tumors [40], and using the same conditional knockout allele of EGFR used here, we also confirmed that the EGFR-independent intestinal and colonic tumors are larger [41], highlighting the unique role of ERBB3-dependent signaling in regulating tumor growth.

To elucidate the mechanism underlying the differentially effect of ERBB3 loss on polyp number and size, we investigated cell proliferation and death in polyps and adjacent normal epithelia. Similar to the previous study on a B6;129 mixed genetic background, a higher number of apoptotic cells were detected in ERBB3-deficient polyps from C57BL/6J-*Apc*^*Min/+*^ mice demonstrating an important role for ERBB3 in tumor cell survival. Transcriptomic analysis also predicted that ERBB3-deficient polyps have decreased levels of p42/44 MAPK activation, which is the predominant mitogenic signal [42]. However, immunohistochemistry results indicate that lack of ERBB3 signaling primarily contributes to tumor growth by increasing cell proliferation as shown in an increase in Ki67 positive cells in ERBB3-deficient polyps. Based on previous studies in lung cancer, PI3K signaling could lead to increase cell proliferation even in the absence of ERBB3 [20,43]. Therefore, the requirement for the ERBB3 signaling pathway in intestinal tumor progression could result from its unique role of linking EGFR signaling to PI3K/AKT, thus activating the PI3K pathway to promote cell proliferation. EGFR can also activate PI3K/AKT through association with the adaptor protein GAB1 in *Apc*^*Min/+*^ polyps [44]. It is possible that PI3K/AKT is activated by EGFR via two mechanisms, association with GAB1 and coupling with ERBB3. Based on previous data and results here, we propose that ERBB3 activation of PI3K/AKT is one of the primary mechanisms supporting *Apc*^*Min/+*^ polyps. The decrease of polyp number and polyp size in the double knockout of *Egfr* and *Erbb3* suggests these two receptors are essential for the development of intestinal polyps.

Unlike the small intestinal polyps in the *Apc*^*Min/+*^ model, epithelial-specific deletion of *Erbb3* in the AOM model did not result in tumor size reduction, although the number of polyps was increased. This difference could be due in part to the fact that *Apc*^*Min/+*^ mice develop polyps by loss of APC, while in the AOM model, tumors are induced by stabilization of beta-catenin. However, recent gene expression profiling shows that these two models are highly similar [45], suggesting that the difference in the route of tumor initiation in the *Apc*^*Min/+*^ and AOM models likely does not contribute to molecular differences resulting in ERBB3 sensitivity. An alternative possibility is that a subset of intestinal polyps can grow independently of ERBB3, similar to previous results observed for EGFR [40,46]

In this study, we observed a profound genetic background-dependent effect on tumorigenesis in the absence of ERBB3. The effect on tumorigenesis of removing ERBB3 may result from its unique link to PI3K/AKT and its downstream effector MAPK. Furthermore, as ERBB3 partners with other ERBB receptor and triggers essential downstream signals, a lack of ERBB3 would abolish EGFR/ERBB3, ERBB2/3, and ERBB3/4 heterodimers simultaneously, which may contribute to its impact on polyp size. The decreased transcript levels of ERBB4 previously reported in ERBB3-deficient intestinal tumors suggests that elevated apoptosis may be due to loss of ERBB3-ERBB4 heterodimers [23], consistent with experiments showing a requirement for ERBB3-ERBB4 heterodimer–dependent AKT pathway activation to prevent CRC cell apoptosis [23]. Consequently, targeting ERBB3 and disrupting heterodimer formation, or using antibodies that inhibit ERBB3 heterodimerization with other ERBBs, may be more efficient than targeting individual receptors, but with the consequence of increased intestinal proliferation and polyp formation in some patients. Our study highlights the importance of regulators of intestinal tumor progression that are dependent on the ERBB3 signaling pathway. It will be important to determine whether ERBB3-dependent signaling also contributes in a genetic background-dependent manner to tumorigenesis in other cancers such as breast, lung, and prostate cancers, where PI3K/AKT is also strongly implicated. These results have important clinical implications that could improve precision applications ERBB3 inhibitor therapy.

## Materials and methods

### Ethics statement

All animal procedures were approved by the Institutional Animal Care and Use Committee at Texas A&M University conforming to the Guide for the Care and Use of Laboratory Animals.

### Animal experiments

All animal studies were maintained, and protocols followed, in accordance with Texas A&M University Institution Animal Care and Use Committee guidelines. Mice were obtained from The Jackson Laboratory (C57BL/6J (B6)-*Apc*^*Min/+*^) and NCI-Frederick (B6;D2-Tg(Vil-Cre)20Syr) and maintained hemizygous on the C57BL/6J background. *Tg(Vil1-Cre)* was selected since it results in extensive target gene deletion throughout the intestinal and colonic epithelia with little expression elsewhere and was used in previous studies with EGFR and ERBB3 [23,41]. *Erbb3*^*tm1*.*1Dwt*^ (*Erbb3*^*f*^) and *Egfr*^*tm1Dmt*^ (*Egfr*^*f*^) mice were maintained on the C57BL/6J background. All alleles were also crossed to the 129S1/SvImJ background and intercrossed to make the B6129 hybrid background. The B6;129 mixed background was reproduced by generating F3-F5 advanced intercrosses. Mice were housed five per cage, fed Purina Mills Lab Diet 2919, and maintained at 22° under a 12-hr light cycle. Mice were euthanized by CO_2_ asphyxiation for tissue collection.

### Genotyping

Mice were genotyped for the *Apc*^*Min*^ allele as previously described [40]. Cre transgenic mice were identified using PCR with cre-S1, 5-gtgatgaggttcgcaagaac and cre-AS1, 5-agcattgctgtcacttggtc primers producing a 278-bp PCR product. Mice were genotyped for the *Egfr*^*tm1Dmt*^ allele as previously described [47] and for the *Erbb3*^*tm1*.*1Dwt*^ allele using B3-F, 5’- TCCAGCGTGGAAAAGTTCAC; and B3-R, 5’- AAGCCTTCTCTATGGAAAGTG.

### Azoxymethane (AOM) carcinogenesis

A single lot of AOM was obtained from Sigma-Aldrich (St. Louis, MO) in 100 mg isovials and stored at -80°C. Each 100 mg vial was resuspended in 2 ml phosphate-buffered saline (PBS) and individual 250 ul aliquots stored at -80°C until use. A working stock of 1.25 mg/ml AOM was made by diluting individual 250 ul aliquots into 10 ml of saline (0.9% NaCl). Three-month-old mice were injected intraperitoneal (IP) 10 mg AOM per kg body weight once a week for 4 weeks as described [48,49]. Age-matched controls were injected with saline.

### Tissue collection

The small intestine and colon were removed at three months of age from *Apc*^*Min/+*^ mice or colons five months after last carcinogen dose from AOM-treated mice. Small intestine was cut into quarters, and each segment was gently flushed with PBS to remove fecal material, cut longitudinally, and splayed flat. Representative tumors were scored before bisecting under a dissecting microscope. One half was used for molecular analysis; the other half was fixed for histological analysis, or snap-frozen for use in cryo-sectioning.

### Macroadenoma counts

Tumor numbers were counted and diameters measured along the entire length of the small intestine and colon with a dissecting microscope and in-scope micrometer at 5x magnification without knowledge of genotype by the investigator. Tumors approximately 0.3 mm in diameter were the smallest that could be counted. Changes in tumor growth rate across genotypes were estimated based on tumor size at date of euthanasia. Tumors were also scored based on location along the intestinal tract. Averages and standard deviations for all polyp counts and sizes are provided (S1 Table) as well as representative whole-mount images (S3 Fig). Approximately 5% of representative polyps were verified by histology.

### Histology and immunohistochemistry

Samples from the *Apc*^*Min/+*^ model were fixed in 10% neutral buffered formalin at 4°C overnight before embedded in paraffin and collecting 7 μm sections every 50 μm for staining. Immunohistochemical procedures were performed as described [50]. Colon tumors were dissected, fixed in 4% paraformaldehyde, and embedded in paraffin before cutting 10 μm sections. After antigen-retrieval by boiling for 20 min in citrate buffer, pH 6.0, sections were treated with 0.3% hydrogen peroxide in PBS for 30 min, washed in PBS, blocked in 3% specific serum and 0.1% Triton X- 100 in PBS, and then incubated with primary antibodies and HRP-conjugated specific anti-rabbit secondary antibody (Vector Laboratories, Inc). DAB peroxidase substrate kit (Vector Laboratories, Burlingame, CA) was used to detect antigen-antibody complexes according to the manufacturer’s protocol. Normal and tumor samples were randomly chosen by blinded personnel, and the samples were then confirmed via hematoxylin and eosin staining. Proliferation (Ki67, ab15580-Abcam) and apoptosis (TUNNEL Assay, ab206386-Abcam) was assessed on five samples of each genotype (with and without ERBB3) on the C57BL/6J background. Cells were scored as positive when nuclear staining was evident. Normal tissue was defined as areas where crypts were well orientated. In tumor regions, the total number of cells and all positively stained cells in the core of the tumor were counted using Fiji ImageJ2 [51] and expressed as a percentage of the total number of cells counted.

For EGFR IHC, 5 μM sections were taken and antigen retrieval performed by boiling in sodium citrate buffer pH 6.0 for 20 minutes. Sections were blocked in TBS plus 10% normal goat serum and 1% BSA for 1 hour before being incubated at 4°C overnight with primary antibody (1:100, ab5652-Abcam) diluted in TBS plus 1% BSA. Sections were then treated with 0.3% hydrogen peroxide in TBS for 15 minutes before incubation with HRP-conjugated secondary antibody (1:2000, ab205718-Abcam) diluted in TBS plus 1% BSA for 1 hour. Antigen-antibody complexes were detected with ImPACT DAB peroxidase substrate (Vector Laboratories SK4105) according to the manufacturer’s protocol. Tissues were counterstained with hematoxylin for 5 minutes before imaging.

### Transcriptomic analysis

Three sequencing runs were performed to sequence 56 samples on a NextSeq500 by the Texas A&M Institute for Genome Sciences and Society using high output kit v2. A total of 1.5 billion 75 bp single-end reads were checked for adapter sequences and low-quality bases using Trimmomatic [52], resulting in approximately 1.4 billion filtered reads (96%). RNA-Seq reads were aligned to mouse assembly mm10 using HISAT2 version 2.0.5 [53] with an overall mapping rate of approximately 97%. Raw gene counts were generated with feature Counts package [54], while discarding ambiguous read mappings. Normalized read counts and gene expression tests were performed using DESeq2 following recommended guidelines [55]. Ingenuity Pathway Analysis (IPA) was used to analyze differentially expressed genes between groups. All RNAseq data is available at NCBI BioProject ID PRJNA635118.

### Quantitative real time PCR (qRT-PCR)

*Egfr* in normal and tumor tissues from C57BL/6J and B6;129 mixed background mice were analyzed by qPCR as previously [41]. Genes with significant changes in expression between polyps with and without *Erbb3* were identified using ANOVA and select genes confirmed by qRT-PCR. cDNA was synthesized from total RNA from each tumor using the QuantiTect Reverse Transcription Kit (Qiagen 205314). PCR reactions were set up in 96-well plates, and all samples were run in triplicate. Analysis was performed on a LightCycler 96 Thermocycler (Roche) using LightCycler 480 Sybr Green I Master reaction mix. Specific primers were designed to amplify a fragment from genes implicated in the RNAseq analysis (S2 Table).

### Statistics

Nonparametric Mann–Whitney U test was used to analyze polyp data. To compare the statistical difference between two groups, student’s *t* test was used. P-values smaller than 0.05 were considered significant.

## Supporting information

S1 Table Mean and standard deviations for all measurements from Figs 1, 4 and 5. (PDF)

S2 Table qPCR primers for genes evaluated for expression differences between genetic backgrounds. (PDF)

S1 Fig Transcriptomic analysis of ERBB3-deficient tumors using IPA predicts downregulation of MAPK1 signaling pathway. Blue, down-regulated genes; orange, up-regulated genes.
(PDF)

S2 Fig Immunohistochemistry for EGFR. (A) C57BL/6J-*Apc*^*Min/+*^, *Erbb3*^*f/f*^, *Tg(Vil1-Cre)*; (B) B6;129-*Apc*^*Min/+*^, *Erbb3*^*f/f*^, *Tg(Vil1-Cre)*. (C) C57BL/6J-*Apc*^*Min/+*^, *Egfr*^*f/f*^, *Tg(Vil1-Cre)* and (D) B6;129-*Apc*^*Min/+*^, *Egfr*^*f/f*^, *Tg(Vil1-Cre)* are no primary antibody controls. Black bars, 50μm.
(PDF)

S3 Fig Representative whole-mount images of *Apc^Min/+^* intestinal sections from mice with intestinal-specific deletion of *Erbb3* on different genetic backgrounds. Arrows point to representative but not all polyps. (PDF)
