# Supplementary material for: Epithelial-specific ERBB3 deletion results in a genetic background-dependent increase in intestinal and colon polyps that is mediated by EGFR
Source: PLoS Genet. 2021 Nov 29;17(11):e1009931. doi: 10.1371/journal.pgen.1009931 (PMC8659709; doi:10.1371/journal.pgen.1009931)
Supplement: S1 Table — (PDF) [file pgen.1009931.s001.pdf]

Fig. 1 Measurements

Background	Measure	*$Apc^{Min/+}$, $Erbb3^{f/f}$*	*$Apc^{Min/+}$, $Erbb3^{f/f}$, Tg(Vil1-Cre)*
C57BL/6J	Intestinal polyp number	118.8 +/- 29.0	266.1 +/- 55.6
	Colon polyp number	1.2 +/- 1.1	3.0 +/- 0.8
	Duodenum polyp size	2.1 +/- 0.2	1.7 +/- 0.1
	Proximal jejunum polyp size	1.5 +/- 0.1	0.9 +/- 0.1
	Distal jejunum polyp size	0.7 +/- 0.1	0.6 +/- 0.1
	Ileum polyp size	1.0 +/- 0.1	1.0 +/- 0.1
	Colon polyp size	3.2 +/- 0.3	1.0 +/- 0.2
B6129 hybrid	Intestinal polyp number	118.9 +/- 20.9	136.7 +/- 28.0
	Colon polyp number	1.1 +/- 1.1	1.0 +/- 0.7
	Duodenum polyp size	2.1 +/- 0.2	1.2 +/- 0.1
	Proximal jejunum polyp size	1.5 +/- 0.1	0.9 +/- 0.1
	Distal jejunum polyp size	1.1 +/- 0.1	0.7 +/- 0.1
	Ileum polyp size	1.0 +/- 0.1	1.0 +/- 0.2
	Colon polyp size	3.2 +/- 0.3	2.9 +/- 0.2
B6;129 mix	Intestinal polyp number	117.3 +/- 26.1	46.0 +/- 16.3
	Colon polyp number	1.4 +/- 1.0	0.7 +/- 0.1
	Duodenum polyp size	2.1 +/- 0.2	0.9 +/- 0.1
	Proximal jejunum polyp size	1.5 +/- 0.1	0.8 +/- 0.1
	Distal jejunum polyp size	1.1 +/- 0.1	0.6 +/- 0.1
	Ileum polyp size	1.0 +/- 0.1	0.6 +/- 0.1
	Colon polyp size	3.2 +/- 0.3	1.9 +/- 0.2

Fig. 4 Measurements

Background	Measure	*$Erbb3^{+/+}$, $R26R^{f/f}$, Tg(Vil1-Cre)*	*$Erbb3^{f/f}$, $R26R^{f/f}$, Tg(Vil1-Cre)*
C57BL/6J	Colon polyp number	1.7 +/- 1.9	4.2 +/- 2.4
	Colon polyp size	3.9 +/- 0.1	3.8 +/- 0.1

Fig. 5 Measurements

Background	Measure	*$Apc^{Min/+}$, $Egfr^{f/f}$, $Erbb3^{f/f}$*	*$Apc^{Min/+}$, $Egfr^{f/f}$, $Erbb3^{f/f}$, Tg(Vil1-Cre)*
C57BL/6J	Intestinal polyp number	118.1 +/- 21.4	9.0 +/- 3.6
	Intestinal polyp size	1.9 +/- 0.1	0.4 +/- 0.1
