# Supplementary material for: Epithelial-specific ERBB3 deletion results in a genetic background-dependent increase in intestinal and colon polyps that is mediated by EGFR
Source: PLoS Genet. 2021 Nov 29;17(11):e1009931. doi: 10.1371/journal.pgen.1009931 (PMC8659709; doi:10.1371/journal.pgen.1009931)
Supplement: S2 Table — (PDF) [file pgen.1009931.s002.pdf]

Gene symbol	Gene name	Gene function	Forward (sequence 5' → 3')	Reverse (sequence 5' → 3')
**ErbB family members**				
Egfr	Epidermal Growth Factor Receptor	ErbB tyrosine kinase receptor	GCATCATGGGAGAGAACAACA	CTGCCATTGAACGTACCCAGA
Erbb3	Epidermal growth factor receptor 3	ErbB tyrosine kinase receptor	TCTGCATTAAAGTCATCGAGGAC	CAGCCGTACAATGTGGGCAT
**RNA-sequencing validation**				
Fas	TNF receptor superfamily member 6	Identical protein binding	TATCAAGGAGGCCCATTTTGC	TGTTTCCACTTCTAAACCATGCT
Tcf3	Transcription factor 3	DNA-binding transcription factor activity	GGGTGCCAGCGAGATCAAG	ATGAGCAGTTTGGTCTGCGG
IL15	Interleukin 15	Cytokine activity	ACATCCATCTCGTGCTACTTGT	GCCTCTGTTTTAGGGAGACCT
Apoe	Apolipoprotein E	Protein homodimerization activity	CTGACAGGATGCCTAGCCG	CGCAGGTAATCCCAGAAGC
Socs3	Suppressor of cytokine signaling 3	Negative regulation of cytokines that signal through the JAK/STAT pathway	ATGGTCACCCACAGCAAGTTT	CTGGAGGCGGCATGTAGTG
**Reference genes**				
Actb	Beta Actin	Cytoskeletal structural protein	GGCTGTATTCCCCTCCATCG	CCAGTTGGTAACAATGCCATGT
Gapdh	Glyceraldehyde3 -phosphate dehydrogenase	Glycolysis pathway enzyme	AGGTCGGTGTGAACGGATTTG	GGGGTCGTTGATGGCAACA
