# Supplementary figures and images for: Epithelial-specific ERBB3 deletion results in a genetic background-dependent increase in intestinal and colon polyps that is mediated by EGFR
Source: PLoS Genet. 2021 Nov 29;17(11):e1009931. doi: 10.1371/journal.pgen.1009931 (PMC8659709; doi:10.1371/journal.pgen.1009931)

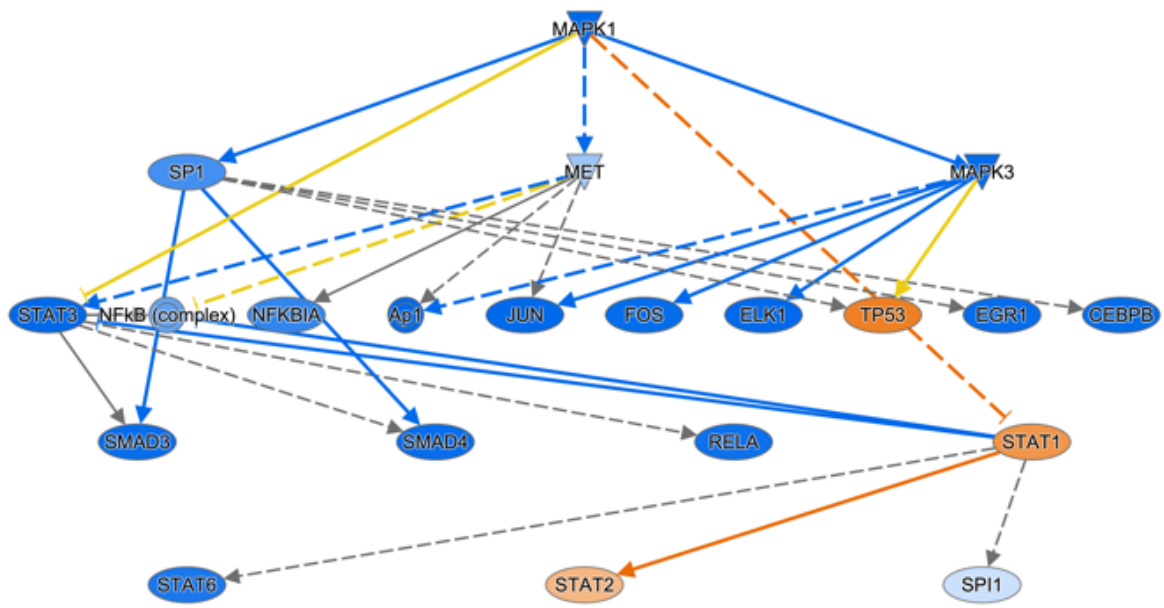

Supplement: S1 Fig — Blue, down-regulated genes; orange, up-regulated genes. (PDF) [file pgen.1009931.s003.pdf]

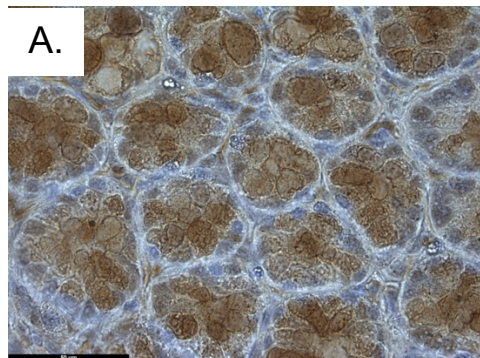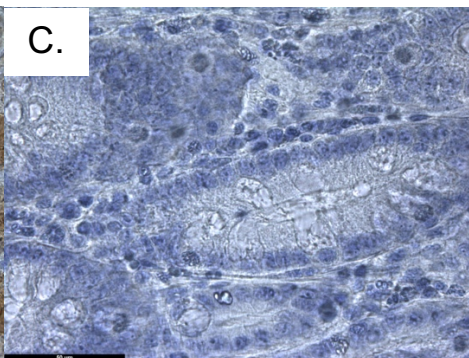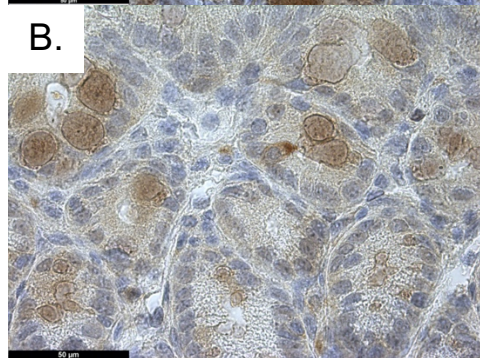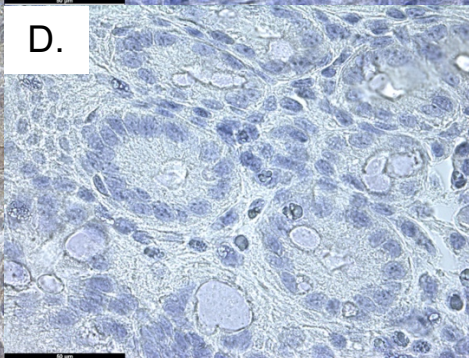

Supplement: S2 Fig — (A) C57BL/6J-ApcMin/+, Erbb3f/f, Tg(Vil1-Cre); (B) B6;129-ApcMin/+, Erbb3f/f, Tg(Vil1-Cre). (C) C57BL/6J-ApcMin/+, Egfrf/f, Tg(Vil1-Cre) and (D) B6;129-ApcMin/+, Egfrf/f, Tg(Vil1-Cre) are no primary antibody controls. Black bars, 50μm. (PDF) [file pgen.1009931.s004.pdf]

Supplementary Figure 3

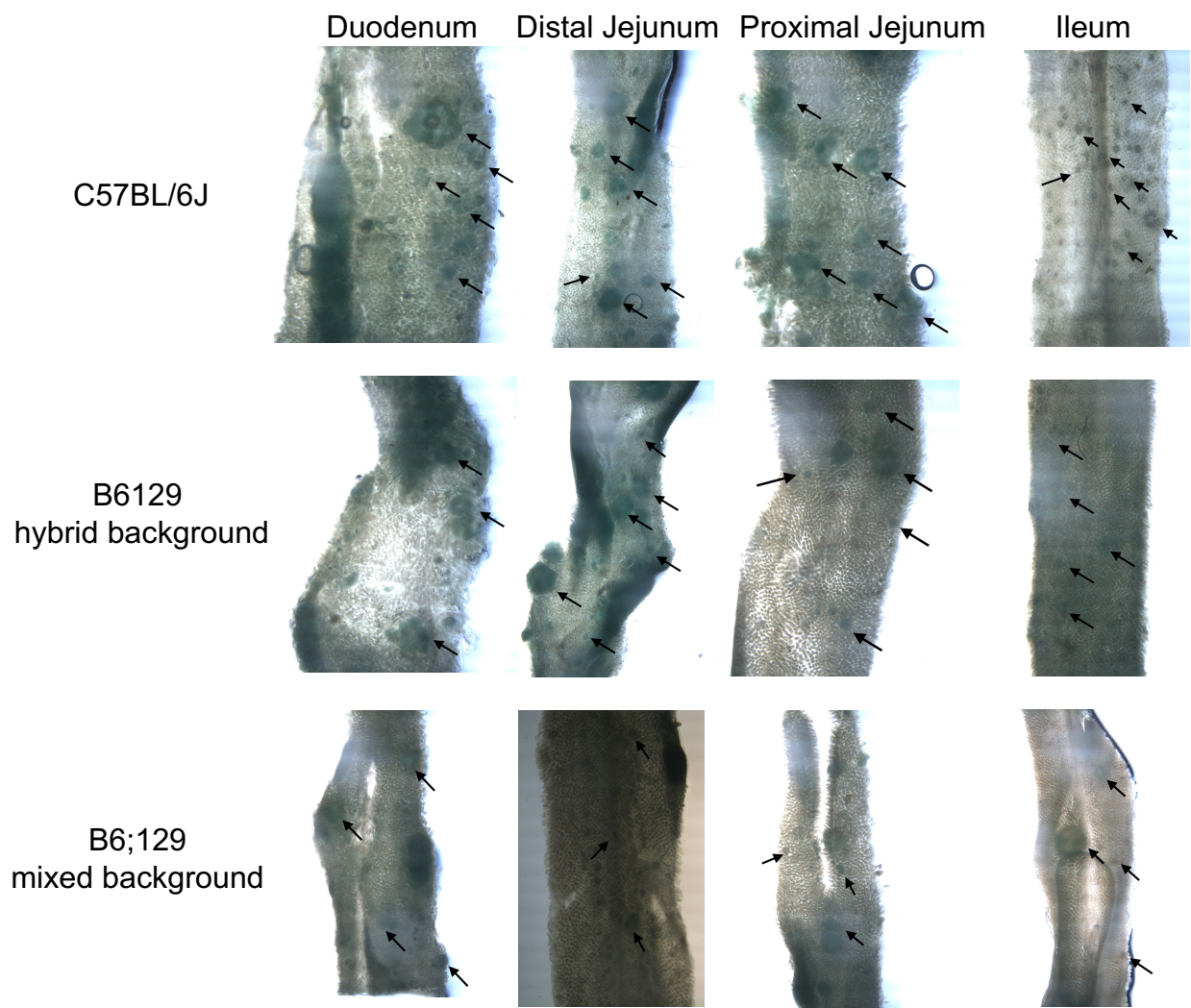

Supplement: S3 Fig — (PDF) [file pgen.1009931.s005.pdf]
